# Baseline Assessment of Mesophotic Reefs of the Vitória-Trindade Seamount Chain Based on Water Quality, Microbial Diversity, Benthic Cover and Fish Biomass Data

**DOI:** 10.1371/journal.pone.0130084

**Published:** 2015-06-19

**Authors:** Pedro M. Meirelles, Gilberto M. Amado-Filho, Guilherme H. Pereira-Filho, Hudson T. Pinheiro, Rodrigo L. de Moura, Jean-Christophe Joyeux, Eric F. Mazzei, Alex C. Bastos, Robert A. Edwards, Elizabeth Dinsdale, Rodolfo Paranhos, Eidy O. Santos, Tetsuya Iida, Kazuyoshi Gotoh, Shota Nakamura, Tomoo Sawabe, Carlos E. Rezende, Luiz M. R. Gadelha, Ronaldo B. Francini-Filho, Cristiane Thompson, Fabiano L. Thompson

**Affiliations:** 1 Institute of Biology and SAGE-COPPE, Federal University of Rio de Janeiro (UFRJ), Rio de Janeiro, Brazil; 2 Rio de Janeiro Botanical Garden Research Institute (IP-JBRJ), Rio de Janeiro, Brazil; 3 Institute of Marine Sciences, Federal University of São Paulo (UNIFESP), Santos, Brazil; 4 Department of Ichthyology, California Academy of Sciences, San Francisco, CA, United States of America; 5 Ecology and Evolutionary Biology, University of California Santa Cruz, CA, United States of America; 6 Department of Oceanography and Ecology, Federal University of Espírito Santo, Vitória, Espírito Santo, Brazil; 7 Associação Ambiental Voz da Natureza, Av Cel. Schwab Filho 104/501, Vitória, Espírito Santo 29050–780, Brazil; 8 Division of Metrology Applied to Life Science (DIMAV), National Institute of Technology, Quality and Metrology (INMETRO), Rio de Janeiro, Brazil; 9 Laboratory of Genomic Research on Pathogenic Bacteria, International Research Center for Infectious Diseases, Research Institute for Microbial Diseases, Osaka University, Suita, Japan; 10 Department of Infection Metagenomics, Genome Information Research Center, Research Institute for Microbial Diseases, Osaka University, Suita, Japan; 11 Laboratory of Microbiology, Faculty of Fisheries Sciences, Hokkaido University, 3-1-1 Minato-cho, Hakodate 041, Japan; 12 Environmental Sciences Laboratory (LCA), Universidade Estadual do Norte Fluminense (UENF), Campos dos Goytacazes, Brazil; 13 National Laboratory for Scientific Computing (LNCC), Petrópolis, Rio de Janeiro, Brazil; 14 Department of Environment and Engineering, Federal University of Paraíba, Rio Tinto, Brazil; Biodiversity Research Center, Academia Sinica, TAIWAN

## Abstract

Seamounts are considered important sources of biodiversity and minerals. However, their biodiversity and health status are not well understood; therefore, potential conservation problems are unknown. The mesophotic reefs of the Vitória-Trindade Seamount Chain (VTC) were investigated via benthic community and fish surveys, metagenomic and water chemistry analyses, and water microbial abundance estimations. The VTC is a mosaic of reef systems and includes fleshy algae dominated rhodolith beds, crustose coralline algae (CCA) reefs, and turf algae dominated rocky reefs of varying health levels. Macro-carnivores and larger fish presented higher biomass at the CCA reefs (4.4 kg per frame) than in the rhodolith beds and rocky reefs (0.0 to 0.1 kg per frame). A larger number of metagenomic sequences identified as primary producers (e.g., Chlorophyta and Streptophyta) were found at the CCA reefs. However, the rocky reefs contained more diseased corals (>90%) than the CCA reefs (~40%) and rhodolith beds (~10%). Metagenomic analyses indicated a heterotrophic and fast-growing microbiome in rocky reef corals that may possibly lead to unhealthy conditions possibly enhanced by environmental features (e.g. light stress and high loads of labile dissolved organic carbon). VTC mounts represent important hotspots of biodiversity that deserve further conservation actions.

## Introduction

Seamounts occur in all ocean basins and are one of the earth’s largest biomes, with at least 125,000 large seamounts occupying nearly 30x10^6^ km^2^, an area which exceeds that of the continental shelves [1, 2]. However, less than 200 seamounts have been surveyed, with most of the investigations covering deep (>500 m depth), aphotic environments [3]. Thus, mesophotic environments (approximately 30–150 m deep) are significantly understudied [4]. Mesophotic communities may be less impacted by the natural and anthropogenic stressors that act on shallow reef systems [5, 6].

Studies on the (microbial) diversity and ecosystem functioning of seamounts are scarce, and major knowledge gaps persist [7]. Studies focused on habitat heterogeneity, biodiversity and connectivity, human impacts, recovery rates, predictive modeling and ecological risk assessments have been recommended to close these knowledge gaps [7]. A limited number of taxonomic assessments based on 16S rRNA sequences have been conducted in deep zones (>500 m) and on volcanically active seamounts [8], but there is no information on the metabolic diversity of euphotic/mesophotic seamounts. In addition, seamounts are threatened by fishing and mining activities because of the presence of abundant fish and extensive deposits of rare metals and carbonate near their peaks [9–11]. Considering the threat levels and biological and ecological relevance of seamounts, an understanding of the core functional aspects of seamounts is crucial.

**Fig 1:**
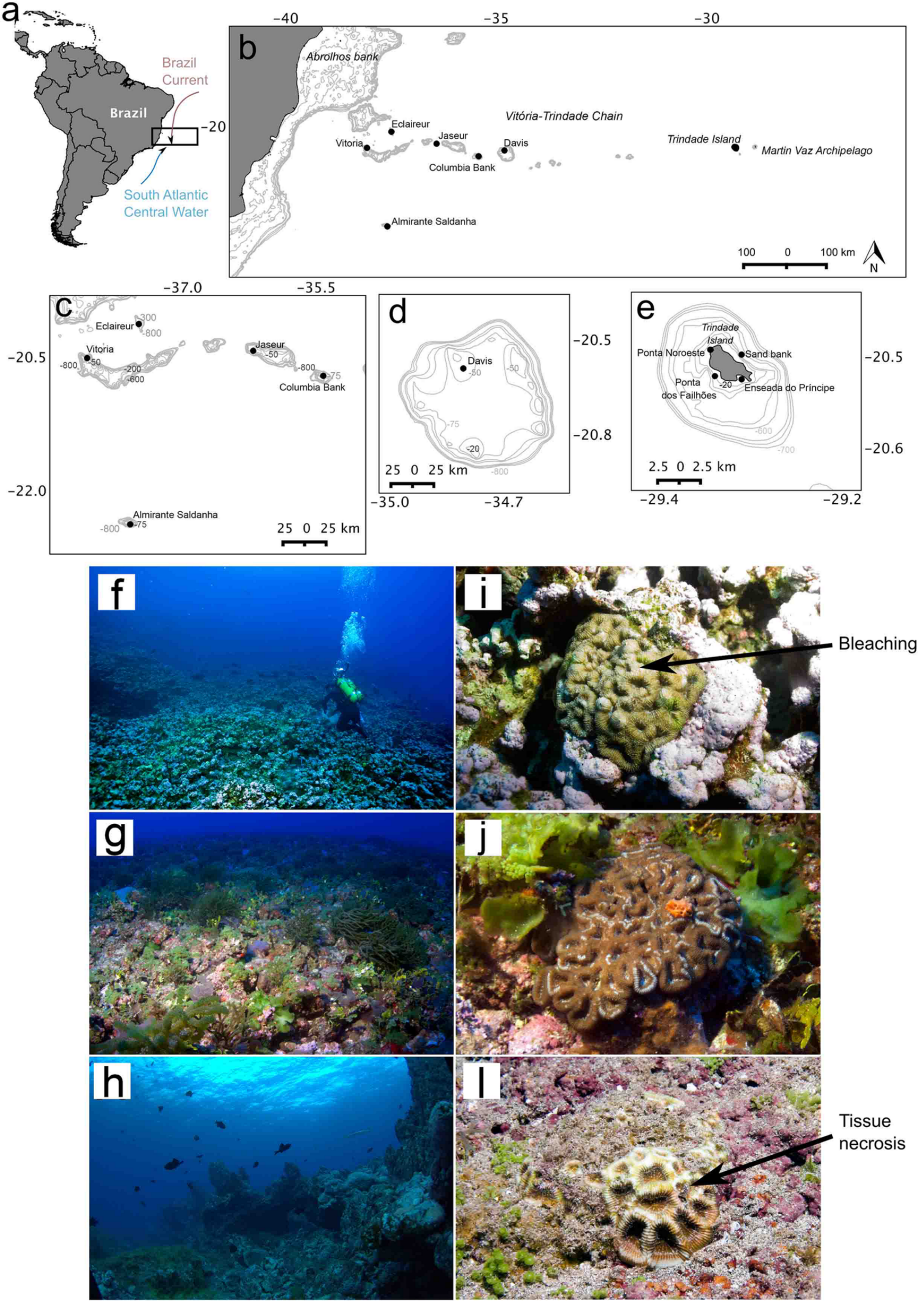
Study area. Data on benthic fish, microbes and nutrients were collected for the Vitoria-Trindade Chain and Trindade Island. Detailed information on the samples and sites can be found in Table 1. (a) Main currents and location of VTC along the Brazilian coast; (b) sampling sites location along the VTC; (c) details of Vitoria, Eclaireur, Jaseur, Columbia Bank and Almirante Saldanha seamounts; (d) details of Davis Seamount sampling site; (e) details of Trindade Island sampling sites; (f) Davis Seamount (CCA reef, Group 1); (g) Jaseur Seamount (fleshy algae dominated rhodolith beds, Group 2); (h) Trindade Island (turf algae dominated rocky reef, Group 3); corals from (i) CCA reef, (j) rhodolith bed and (l) rocky reef. The maps were generated using Qgis software [68]. Photo credit: Ronaldo Francini-Filho.

The Vitória-Trindade Chain (VTC) consists of 11 heterogeneous seamounts with summits reaching euphotic and mesophotic zones (10–110 m depth), and it extends 1,150 km off the eastern coast of Brazil to Trindade Island and the Martim Vaz Archipelago in the Atlantic Ocean (Fig 1B). These seamounts appear to be hotspots of bacterial and primary productivity [12, 13] and contain concentrations of large stocks of commercially important fish [14]. At present, quantitative information on the fish, benthic and microbial assemblages of the VTC is not available. Although distant from the coast, the islands only contain a relatively small amount of endemic species (<9.6%) within the reef-associated biota [15]. Therefore, it has been hypothesized that the VTC summits facilitate connectivity between the coral, fish and gastropod fauna of the continental shelf, Trindade Island and Martim Vaz Archipelago [15–17].

Recently, structural reefs built by crustose coralline algae (CCA) were found at the VTC summits [18]. These reefs were referred to as “reef oases” because their fish fauna is richer than that of the surrounding flat environments. These reef patches may be a critical habitat in the seamount chain because they could serve as stepping-stones to connect several reef-associated organisms that occur in the isolated islands (Trindade and Martim Vaz Archipelago). However, it is not fully known how heterogeneous are the VTC seamounts. In the present study, we tested the following hypotheses for the VTC seamounts features (i.e., CCA reefs, rhodolith beds and rocky reefs): H1) the benthic assemblages are different among the different seamounts features; H2) the fish assemblage composition and biomass are different among the different seamounts features; H3) the taxonomic and H4) the functional composition of the water metagenomes are different among the different VTC seamounts features; H5) the taxonomic and H6) the functional composition of the coral holobionts metagenomes are different among the different VTC seamounts features; H7) the coral holobionts are genetically connected among the different VTC seamounts features. Using a broad quantitative approach, we characterized the mesophotic reef environments of VTC by conducting benthic and fish assemblage assessments, measuring water quality (chemical, biological and microbiological parameters) and analyzing the metagenomes of seawater and corals. By comparing the corals, metagenomes and health status of the seamounts and Trindade Island, we were able to assess the possible effects of the environment on coral health. Our data provide a holistic view of the seamount chain and support the need to designate these communities as marine protected areas.

## Material and Methods

### Study region and sampling procedures

The VTC seamounts are distributed along a fracture zone transversal to the Mid-Atlantic Ridge between 19° and 21°S (Fig 1A and 1B). The eleven VTC seamounts form a 1.100 km west-east chain that culminates in the Trindade and Martim Vaz Islands, and the seamounts are isolated from the continental margin and from each other by deep waters (2,500–4,500 m) and vast distances (50 to 250 km). With the exception of the deeper Columbia Seamount (90 m, highly complex topography), the remaining VTC seamounts have summits that are remarkably flattened, largely covered by rhodolith beds [19], and have average depths of 50 m with sparse, shallower outcrops reaching depths of 20 m.

Superficial circulation in the VTC is dominated by the Brazil Current (BC), which flows south along the eastern South American shelf from approximately 13 to 38°S [20]. The BC flows predominantly through the two passages to the west of Vitória Seamount (50–60 cm.s^-1^) rather than through the more offshore passages to the east. A cyclonic gyre seaward of the BC is attributed to the southernmost meanders of the South Equatorial Current, which are reflected northwards by the VTC [21]. At the southern part of the chain, the warm (18–28°C) and oligotrophic BC interacts frequently with the colder South Atlantic Central Water (SACW) flowing below the mixed layer to create the Vitória Eddy (oxygen and nutrient rich waters) [22, 23]. Mesoscale circulation in the VTC region is still poorly understood, but the topographical complexity of the VTC induces SACW upwelling driven by eddies, Ekman pumping and tidal current amplification [24].

Benthic and fish assemblages, water quality (metagenomic, chemical and biological inventory analyses) and *Mussismilia hispida* corals (metagenomic analyses for six specimens) were sampled by SCUBA divers using trimix in March 2009 and February 2011. Differences in the average sea surface temperature (SST) and chlorophyll *a* concentration between March 2009 and February 2011 were 0.6°C and 0.005 mg/m^3^, respectively, in the study area [Lat (-24, -16.45), Long (-40, -25)] (SST data source: MODIS-Terra; chlorophyll *a* data source: MODIS-Aqua). Based on these slight differences, we used samples from the two years as replicates for each site. Sampled sites included the carbonate summit of six seamounts and four rocky reef sites (basaltic) at Trindade Island (Fig 1). At all sites, an approximately 200 m^2^ area was covered. The number of replicates and experimental design details are summarized in S1 Table. All of the coral colonies were preserved in liquid nitrogen at the Laboratory of Microbiology (Federal University of Rio de Janeiro, Brazil). Details on the analyzed colonies are included in S1 Table. Sampling was authorized by the Brazilian Environmental Agency, Instituto Chico Mendes de Conservação da Biodiversidade (SISBIO 21811–1).

### Benthic cover surveys

To recognize the main topographic features of the seamount peaks, we used side-scan sonar (EdgeTech 4100, 100–500 kHz, West Wareham, Massachusetts, USA) across a linear extent of 110 km with 400 m swaths covering the Trindade Island shelf and upper slope as well as the summits of seamounts Jaseur and Davis. Sonograms were processed with SonarWiz Map4 V.4.02 (Chesapeake Technology Inc, Mountain View, California, USA). Benthic cover was estimated following previously described procedures [25, 26]. Ten 0.7 m^2^ photoquadrats were randomly placed at each site, and the percent cover was estimated using Coral Point Count with Excel Extension software (CPCe) [27] and 15 randomly distributed points per photograph (225 points per quadrat). Organisms below each point were identified to the following major benthic categories: turf algae (subdivided into *Jania* plus *Amphiroa* plus other small filamentous algae and the cyanobacteria *Lyngbya* sp.), sand, sponge, fleshy algae [28], and coral (mainly *Siderastrea* sp., *Montastrea cavernosa* and the Brazilian endemic *M*. *hispida*). For each coral colony, the health status was assessed based on three categories: vitality, presence of tissue necrosis, and bleaching. The reference samples of algae were deposited in the collection of the Rio de Janeiro Botanical Garden Herbarium (RB).

### Fish assemblage surveys

Fish assemblages were assessed from video records using a remote operated vehicle (ROV), Seabotix LBV 150S2 (San Diego, California, USA) equipped with lights and a color video camera and pair of scaling lasers spaced 5 cm apart (used to estimate fish sizes), and video cameras handled by divers using standard SCUBA gear (<40 m deep) and mixed-gas techniques (trimix) in open systems (>40 m deep). The fish samples were obtained at the same sites where the water samples were obtained at depths ranging from 25 to 63 m. Both the ROV and diver-based recordings were performed with slow movement (not static) near the bottom (approximately 1 m) and focused on all of the available habitats (e.g., rhodoliths, interface, reefs, water column, etc.) to record the entire reef fish community. Fish counts were performed from frames of video footage recorded every 10 seconds. A total of 546 frames recorded from the ROV and divers were used (180 on Trindade Island, 275 on Davis Seamount and 91 on Jaseur Seamount). The size of the fish (total length [TL]) was visually estimated using the laser scale, and individuals were classified into 10 cm size classes. Fish biomass was estimated using length-weight relationships [29]. When no relationship was available for a species, an equation from similarly sized congeners was applied. Fish species were assigned to one of the following trophic guilds based on adult diet data [30]: herbivores, invertivores, macro-carnivores and omnivores. Fish results are presented as the relative abundance (number of fish from a category [size or trophic guild]/total number of fish from the site) and biomass (kg/frame).

### Chemical and biological inventories

Divers collected water samples at the benthic boundary layer (up to 1 m from the bottom) using two 10 L Niskin bottles (Table 1). At least three replicate samples were obtained for each parameter in each of the eight locations. Chlorophyll *a*, dissolved organic carbon (DOC), inorganic nutrients, phosphorus and nitrogen concentrations and microbial abundance were determined following previously described methods [12, 31, 32]. Chlorophyll *a* samples were collected using positive pressure filtration with 2 L of water. The filters (cellulose HAWP, Millipore, Darmstadt, Germany) were extracted overnight in 90% acetone at 4°C and analyzed by spectrophotometry or fluorimetry. For the DOC analysis, 50 mL of filtered seawater (calcinated and weighted Whatman GF/C glass microfiber filter, Sigma-Aldrich, St. Louis, Missouri, USA) was collected and fixed with 100 μL of phosphoric acid. Samples were refrigerated at 4°C until DOC quantification procedures could be conducted at the laboratory. For the inorganic nutrient analyses, 1 L of water was frozen and analyzed in a laboratory using the following methods: 1) ammonia by indophenol, 2) nitrite by diazotization, 3) nitrate by reduction in a Cd—Cu column followed by diazotization, 4) total nitrogen by digestion with potassium persulfate following nitrate determination, 5) orthophosphate by reaction with ascorbic acid, 6) total phosphorous by acid digestion to phosphate, and 7) silicate by reaction with molybdate. The DOC, phosphorus and nitrogen concentrations are shown in Table 1, and the sum of the organic and inorganic P and N are listed as the Total P and Total N, respectively. The microbial abundance was determined from three replicates of seawater per site by flow cytometry with SYBR green (Life Technologies, Carlsbad, California, USA).

**Table 1:** Information on the sampling sites and chemical and biological inventories of seawater samples.

	Group 1 (CCA reef)	Group 2 (Fleshy algae dominated rhodolith beds)						Group 3 (Turf algae dominated rocky reefs)		
Site	Davis ^a^ ^,^ ^c^ ^,^ ^d^	Jaseur ^a^ ^,^ ^b^ ^,^ ^d^	Columbia Bank ^a^	Almirante Saldanha ^a^	Vitoria ^c^ ^,^ ^d^	Eclaireur ^a^	Trindade Shelf ^c^	Ponta do 5 Farilhões ^a^ ^,^ ^c^ ^,^ ^d^	Ponta Noroeste ^a^ ^,^ ^c^ ^,^ ^d^	Enseada do Príncipe ^a^ ^,^ ^c^
**Latitude**	-20.577	-20.485	-20.713	-22.382	-20.514	-20.132	-20.504	-20.530	-20.493	-20.526
**Longitude**	-34.80645	-36.130	-35.421	-37.588	-38.071	-37.492	-25.355	-29.330	-20.345	-29.31035
**Depth (m)**	40	60	62	66	63	71	50	22	15	20 and 22
**Sampling date**	11-Feb-11	9-Feb-11	10-Feb-11	24-Feb-11	4-Feb-11	8-Feb-11	18-Feb-11	18-Feb-11	18-Mar-09	20-Feb-09 and 21-Feb-09
**Light Classification**	Mesophotic	Mesophotic	Mesophotic	Mesophotic	Mesophotic	Mesophotic	Mesophotic	Euphotic	Euphotic	Euphotic
**P-PO**_**4**_^**3-**^ **(0μM)**	0.14±0 (N = 3)	-	-	-	0.08±0.01 (N = 3)	0.08±0 (N = 3)	0.55±0.01 (N = 3)	0.13±0.01 (N = 3)	0.1±0 (N = 3)	0.09 (N = 6)
**Total P (μM)**	0.25±0 (N = 3)	-	-	-	0.22±0 (N = 3)	0.23±0 (N = 3)	0.72±0.01 (N = 3)	0.29±0.01 (N = 3)	0.28±0.07 (N = 3)	0.27±0.04 (N = 5)
**N-NH**_**3**_ **/NH**_**4**_^**+**^ **(μM)**	<0.05±0 (N = 3)	-	-	-	<0.05±0 (N = 3)	<0.05±0 (N = 3)	<0.05±0 (N = 3)	<0.05±0 (N = 3)	0.49±0.2 (N = 3)	0.4±0.12 (N = 5)
**N-NO**_**3**_^**-**^ **(μM)**	1.43±0.04 (N = 3)	-	-	-	1.24±0.02 (N = 3)	1.27±0.01 (N = 3)	1.13±0.04 (N = 3)	1.17±0.03 (N = 3)	0.54±0.01 (N = 3)	0.7±0.01 (N = 7)
**N-NO**_**2**_^**-**^ **(μM)**	0.05±0.01 (N = 3)	-	-	-	0.03±0 (N = 3)	0.02±0 (N = 3)	0.03±0 (N = 3)	0.05±0 (N = 3)	0.03±0.01 (N = 3)	0.02 (N = 5)
**Total N (μM)**	10.36±0.41 (N = 3)	-	-	-	7.15±0.3 (N = 3)	8.2±0.58 (N = 3)	8.85±1.09 (N = 3)	8.96±0.47 (N = 3)	20.72±1.17 (N = 3)	8.52±1.7 (N = 4)
**Silicate (μM)**	0.76±0.02 (N = 3)	-	-	-	0.85±0.02 (N = 3)	0.77±0.04 (N = 3)	0.93±0.03 (N = 3)	1.09±0.06 (N = 3)	1.24±0.33 (N = 3)	0.9±0.07 (N = 6)
**Salinity (S)**	37.35±0.03 (N = 3)	-	-	-	37.07±0.01 (N = 3)	37.26±0.02 (N = 3)	37.22±0.03 (N = 3)	37.11±0.05 (N = 3)	37.62±0.01 (N = 3)	37.67±0.1 (N = 6)
**Chlorophyll *a* (μg.L** ^**-1**^ **)**	0.12±0.04 (N = 3)	-	-	-	0.18±0 (N = 3)	0.25±0.01 (N = 3)	0.26±0.03 (N = 3)	0.17±0.03 (N = 3)	-	-
**Phaeophytin (μg.L** ^**-1**^ **)**	0.06±0.03 (N = 3)	-	-	-	0.12±0 (N = 3)	0.12±0.01 (N = 3)	0.24±0.01 (N = 3)	0.16±0.03 (N = 3)	-	-
**Bac. Counts (cells.mL** ^**-1**^ **)**	7.66 x10^5^±3.48 x10^4^ (N = 4)	-	-	-	1.09x10^6^±5.74x10^4^ (N = 4)	4.68x10^5^±4.78x10^4^ (N = 4)	1.04x10^6^±1.15 x 10^5^ (N = 4)	9.48x10^5^±9.94 x 10 (N = 4)	-	-
**DOC (μM)**	210±29.17 (N = 2)	-	-	-	203.3±4.17 (N = 2)	190±3.33 (N = 2)	214.17±0 (N = 2)	219.17 (N = 1)	-	-

The table includes chemical and biological data and information on the data collection. Data are presented as the mean±SE.

^a^–benthos assessment

^b^–fish assessment

^c^–water metagenomics

^d^–coral metagenomics.

### DNA extraction, pyrosequencing and sequences analyses

The seawater samples were filtered through Sterivex filter units (0.22 μm, Millipore, Darmstadt, Germany) by positive pressure. A total of 4 L seawater was filtered through each Sterivex filter, and the microbes collected in the filters were preserved with a SET buffer (20% sucrose, 50 mM ethylenediaminetetraacetic acid [EDTA] and 0.5 mM Tris-HCl) in liquid nitrogen until DNA extraction procedures could be conducted at the laboratory. Seawater metagenomic DNA extraction was performed following alkaline lysis as previously described [33]. Collected corals were also stored in liquid nitrogen until DNA extraction procedures could be completed. Cetyltrimethyl ammonium bromide (CTAB) buffer with 100 mM EDTA and a PowerSoil (MO BIO, Carlsbad, California, USA) purification column were used to gather high molecular weight DNA from small fragments of each coral sample (approximately 1 cm^2^) [34]. High quality DNA extracted from the Sterivex filters and corals was sequenced using a pyrosequencing GS FLX Titanium kit (Roche, Basel, Switzerland) [35].

Low quality DNA, duplicates, and short sequences (<100 bp) were removed using PRINSEQ [36]. All of the possible contaminants (e.g., human sequences) were removed using DECONSEQ [37]. The sequences’ assignments were conducted using the MG-RAST server [38] and the following cut-off parameters: expected value less than 1x10^-5^, 60% minimum identity and 15 base pair minimum alignment. Taxonomic annotation was performed using the National Center for Biotechnology Information (NCBI) GenBank database, and functional annotation was completed using the SEED database. To standardize the metagenome sizes, we present the metagenomic data as relative abundances (the number of sequences of a given taxa or subsystem of a metagenome divided by the total number of identified sequences of this metagenome). The data generated during this study are stored on http://marinebiodiversity.lncc.br (package pmeirelles.3.1). Metagenomic data are also available from the MG-RAST server, and their unique identifiers are listed in S2 and S3 Tables.

### Data analyses

All of the abundances (including the benthic and fish abundances) were plotted via ggplot2 and reshape [39, 40], which are both R packages [41]. The nine locations analyzed in this study were separated into three macro-habitat groups based on the percentages of the major benthic categories, side-scan sonar data, ROV data, and diver observations (Fig 1F–1I) to test the hypotheses described bellow. To test the hypothesis that benthic assemblages among the macro habitat groups are different (H1), a permutational multivariate analysis of variance (PERMANOVA) was performed using the function adonis in the vegan R package [42]. To test the hypothesis that the fish assemblage composition and biomass are different among the macro habitat groups (H2), an analysis of variance (ANOVA) with Tukey's honest significant difference (HSD) post-hoc method was performed using the TukeyHSD function in R. To verify if the samples from different macro-habitats group together according to the concentrations of nutrients, chlorophyll *a* and phaeophytin and abundance and diversity of bacteria, a principal component analysis (PCA) was performed using the prcomp function in R. Only samples from the Vitoria, Davis and Trindade Shelf seamounts and from Enseada do Príncipe (Trindade Island) were included in the PCA (see S1 and S2 Tables for sample details) because all of the environmental parameters were collected for these locations. Bacterial diversity indices (Shannon entropy and Shannon evenness [i.e., Hill’s Ratio]) and richness were calculated with the vegan R package [42] using the family taxonomy level. To test the hypothesis that the metagenomic composition (taxonomic and functional) from the water (H3 and H4) and the coral (H5 and H6) metagenomic samples are different among the macro habitat groups, an ANOVA with a Tukey-Kramer post-hoc test, eta-squared effect size statistics and Storey false discovery rate correction for multiple tests were performed using STAMP v.2.0.8 software [43]. P-values of <0.05 were considered statistically significant. To assess the genetic similarity between the water microbiome and *Mussismilia* coral holobionts, the dinucleotide composition of the corals and water metagenomes were compared. Frequency tabulation of the sequence data was performed according to [44] and with homemade Python scripts, and a nonmetric multidimensional scaling (NMDS) analysis of the tabulated data was performed using the metaMDS function in the vegan R package to determine if the samples would group together. To test the hypothesis that the coral holobionts and the water microbiomes are genetic connected, the dinucleotide composition was analyzed among the macro habitat groups (water and corals separately) (H7), a PERMANOVA was performed using the function adonis in the vegan R package. P-values of <0.05 were considered statistically significant.

## Results

### Topography and benthic cover

**Fig 2:**
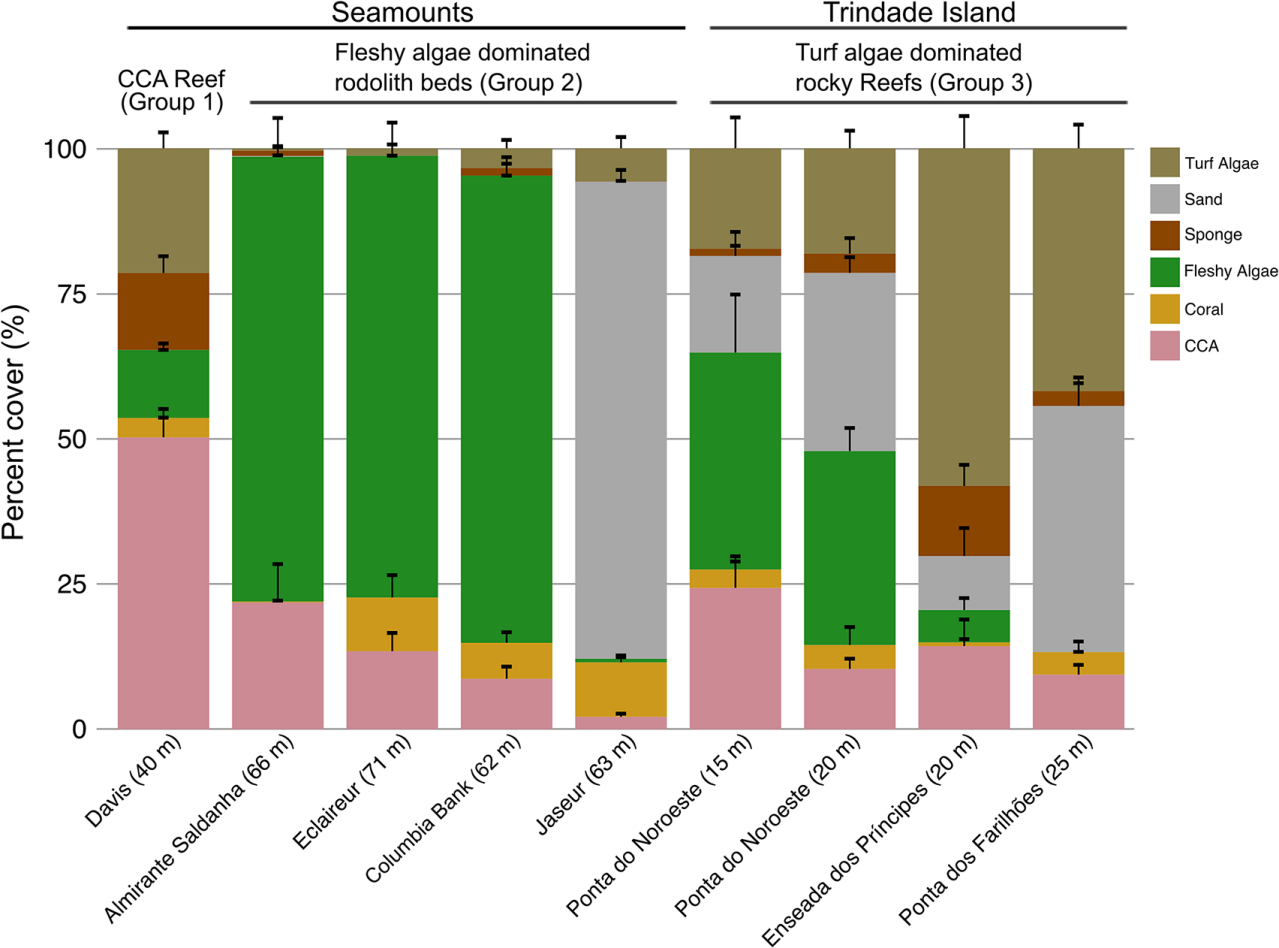
Relative abundance of the major benthic categories for each sampled system. Data presented as the mean±SE.

The side-scan sonar data with high reflectivity and flat topography is confirmed by ROV imagery and reveals that rhodolith beds are the predominant bottom type at the VTC seamount summits and outer insular shelves. The benthic cover was significantly different among the groups (PERMANOVA, p<0.05) (Fig 2 and S3 Table) (H1 confirmed). Group 1 corresponded to CCA reefs consisting of calcareous outcrops of approximately 30 m in height with irregular topography. These CCA reefs were extensive at the Davis Seamount summit but were not recorded at the other seamounts (Fig 1F). Group 2 consisted mainly of flat fleshy algae dominated rhodolith beds, which was the predominant bottom type at all summits and also at the Trindade insular outer shelf (>25 m depths) (Fig 1G). Group 3 consisted of turf algae dominated rocky reefs with an exposed basaltic framework, which was found across the islands’ shores (0–40 m) (Fig 1H), although it did not have significant carbonate accretions from CCA or coral growth.

CCA (50%) and, to a lesser extent, turf (~21%) dominated Group 1, whereas fleshy algae was the most frequent benthic category (~80%) in Group 2. However, Jaseur Seamount was an exception with sand as the dominant benthic category (~80%) (Fig 2). Although Jaseur Seamount had the highest percent of sand cover, a dense rhodolith layer was found underneath; therefore, we were able to classify it into Group 2. Group 3 consisted mainly of fleshy algae (~33–35% at shallow sites) and turf algae (~40–60% at deep sites) (Fig 2). The CCA were identified as *Hydrolithon onkodes*, *Lithophyllum prototypum*, *Peyssonelia* sp., *Phymatolithon masonianum*, and *Spongites* sp., whereas the turf algae consisted mainly of *Jania* sp., *Amphiroa* sp., other small filamentous algae, and the cyanobacteria *Lyngbya*. The most abundant coral species were *Siderastrea* sp., *M*. *cavernosa* and *M*. *hispida*. A marked difference in the health of *M*. *hispida* was observed between mesophotic and shallow environments. Tissue necrosis and bleaching affected >90% of the coral colonies in the shallow waters of Trindade Island and <50% of the coral colonies on Davis Seamount. However, >90% of the coral colonies at the mesophotic sites (rhodolith beds and rocky reefs) were healthy (Fig 1I–1J).

### Fish assemblages

**Fig 3:**
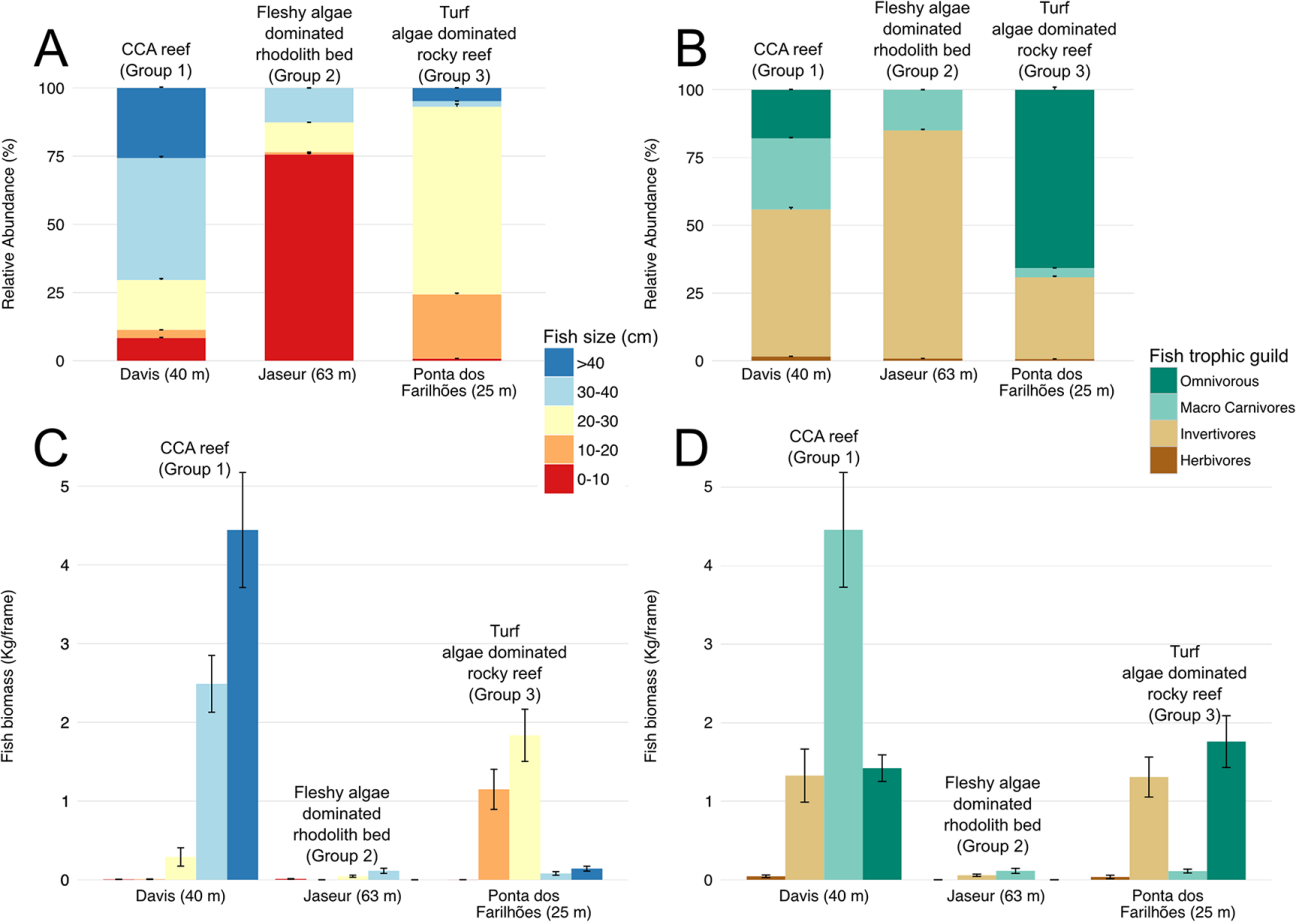
Relative abundance of fish size classes (a) and trophic guilds (b) and biomass of fish size classes (c) and trophic guilds (d). Data are presented as the mean±SE.

CCA reefs (Group 1) had the highest fish biomass compared to fleshy algae dominated rhodolith beds (Group 2) and turf algae dominated rocky reefs (Group 3) (ANOVA, p<0.05) (Fig 3; H2 confirmed). CCA reefs had the highest relative abundance and biomass of larger fish (>40 cm) (~25% and 4.4 kg/frame) (Fig 3A and 3C). At Jaseur Seamount (Group 2), small-sized fish (0–10 cm) had the highest relative abundance (~75%), although medium-sized fish (30–40 cm) had a higher biomass (0.11 kg/frame). Trindade Island (Ponta dos Farilhões) was an intermediate location with the highest relative abundance and biomass of small-sized (10–20 cm; ~25% and 1.15 kg/frame) and medium-sized fish (20–30 cm; ~70% and 1.84 kg/frame). Davis Seamount had a higher relative abundance and biomass of macro-carnivores (~25% and 4.44 kg/frame), whereas Pontas do Farilhões had a higher abundance and biomass of omnivorous fish (~65% and 1.76 kg/frame) (Fig 3B and 3D). Jaseur Seamount had a higher biomass of macro-carnivores (0.11 kg/frame).

### Microbial abundance, diversity and water parameters

Microbial abundance in the water column varied between 4.68x10^5^±4.78x10^4^ and 1.09x10^6^±5.74x10^4^ among the locations (Table 1). Samples from the Trindade Shelf and Davis Seamount (Groups 2 and 1, respectively) had higher bacterial diversity and evenness (Shannon entropy and evenness; see S4 Table). Samples from Trindade Island were the least diverse and most even. The Trindade Shelf (Group 2) had the highest observed orthophosphate concentration (0.55 μM), whereas the Vitoria and Eclareur Seamounts had the lowest values (0.08 μM). Davis Seamount (Group 1) had the highest observed value of nitrate (1.43 μM). The highest observed value of DOC was 219.17 μM at Ponta dos Farilhões (Group 3). Fleshy algae dominated rhodolith bed sites (Group 2) had higher concentrations of chlorophyll *a* (0.18–0.27 μg.L^-1^).

**Fig 4:**
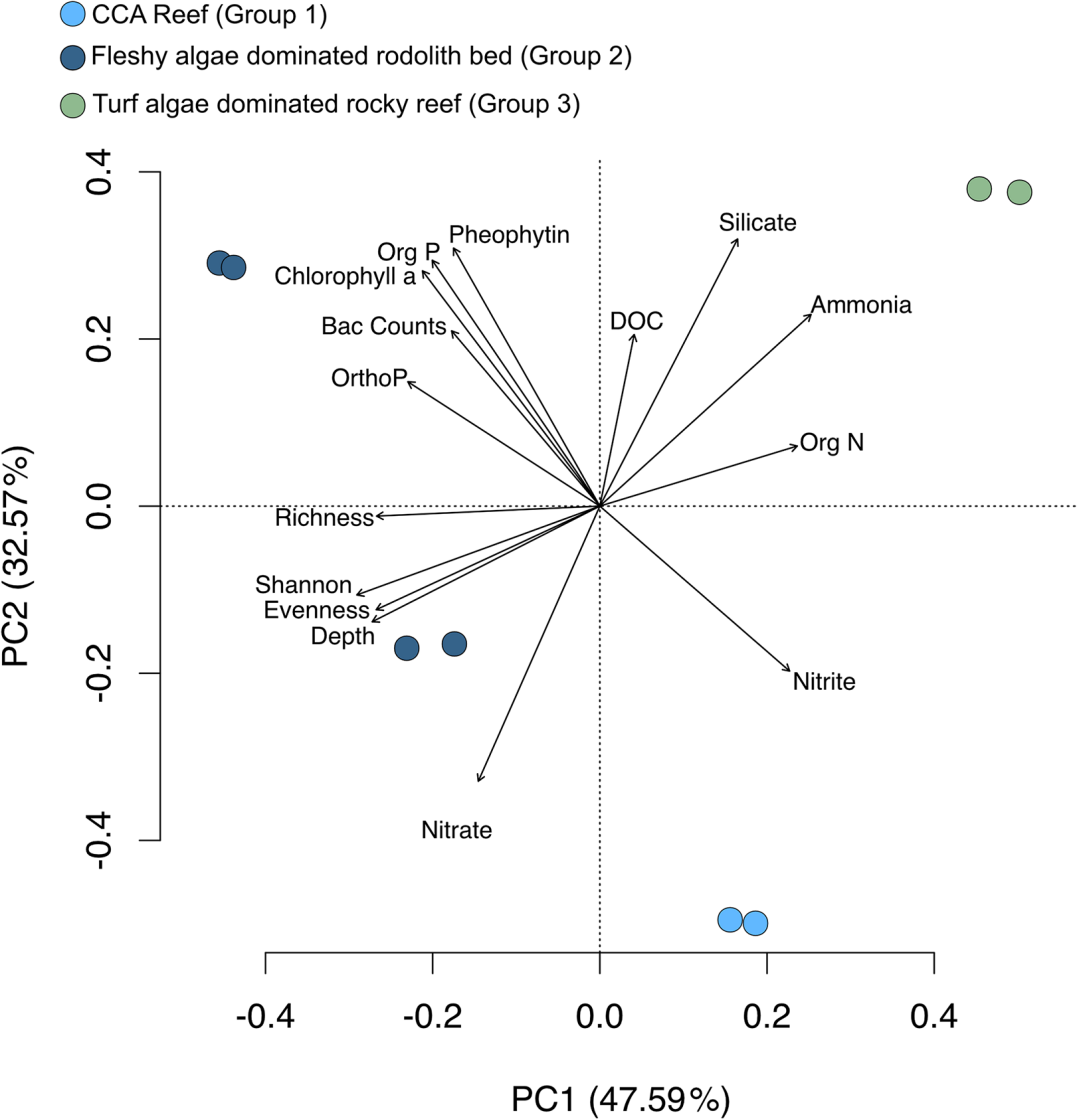
Principal component analysis of water chemical and biological inventories. Abbreviations: Bac Counts–Bacterial counts; DOC–Dissolved Organic Carbon, Org N–Organic Nitrogen, Ortho P–Orthophosphate, Org P–Organic Phosphorous, Richness–Number of bacterial families, Shannon–Shannon entropy index, Evenness–Shannon evenness index (Hill’s Ratio).

The macro-habitat grouping was supported by PCA. The two first axes explained a large proportion of the variation between the samples (PC1 45.74% and PC2 34.34%) (Fig 4). CCA reefs (Group 1) had higher values of nitrite and nitrate. Bacterial counts were highly correlated with organic phosphorous, orthophosphate, chlorophyll *a* and phaeophytin (higher values in rhodolith beds–Group 2). Bacterial family richness and evenness were highly correlated with depth and, to a lesser degree, nitrate values (higher values in rhodolith beds–Group 2). Organic nitrogen, silicate, DOC and ammonia were correlated (higher values in turf algae dominated rocky reefs–Group 3).

### Metagenomic analysis of seawater and *M*. *hispida*

**Fig 5:**
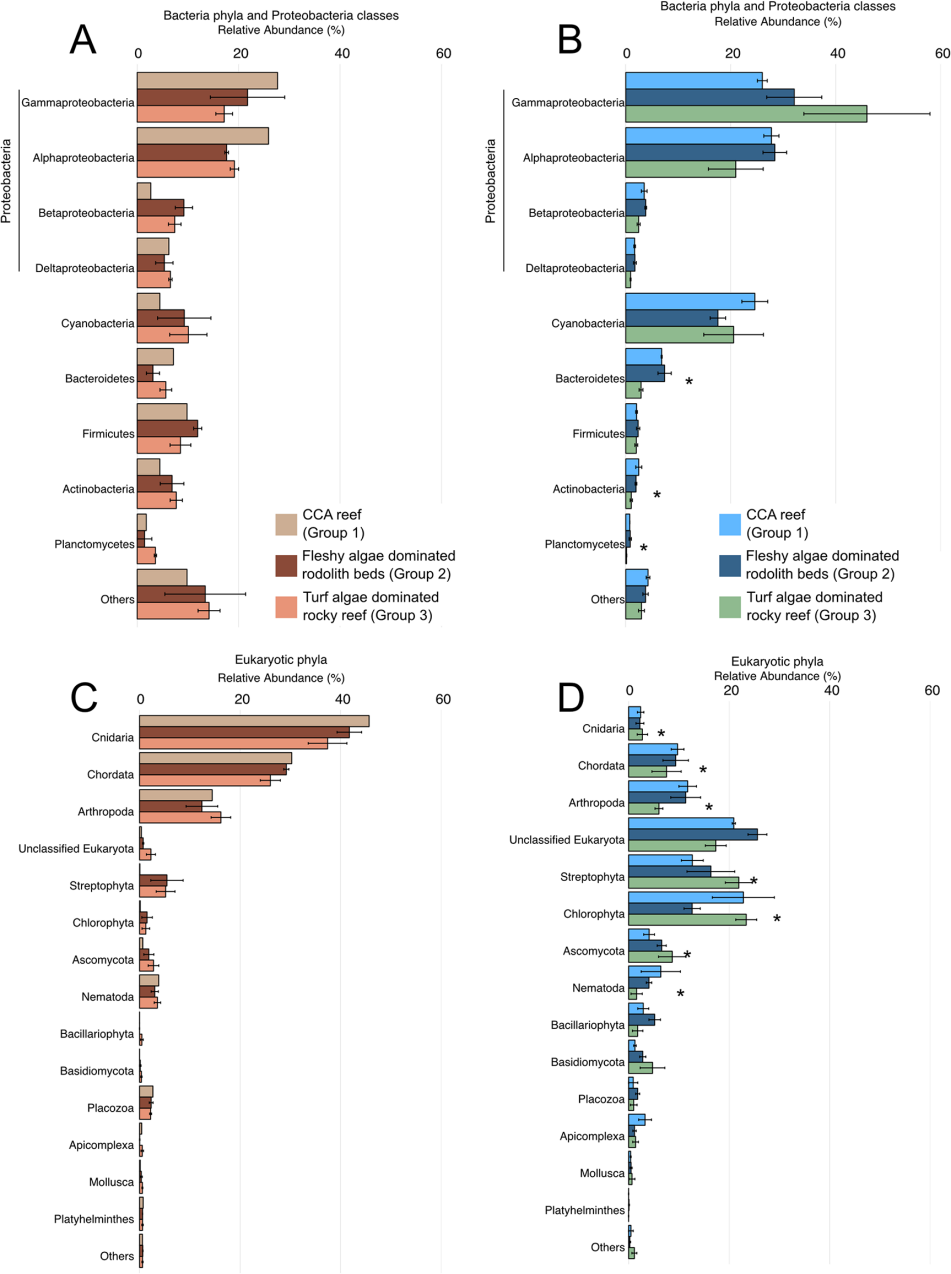
Taxonomic diversity of the metagenomes. Bacterial and proteobacterial phyla relative abundance in corals (a) and water (b). Eukaryotic phyla relative abundance in corals (c) and water (d). Asterisks show significant differences between the different macro-habitats (groups). Data are presented as the mean±SE.

A total of 439,201 high quality metagenomic sequences from 19 samples (nine *M*. *hispida* colonies and ten water samples) were generated with library sizes between 8,182 and 90,559 reads (S2 Table). Median values of seawater metagenomic sequences that were taxonomically and functionally identified were 53.12% and 82.43%, respectively (S2 Table). However, coral metagenomes had only 8.4% and 4.25% of their sequences taxonomically and functionally identified, respectively (S2 Table). Corals had higher median values of identified eukaryotic sequences (72.27%) compared with water metagenomes (2.44%) (Fig 5 and S3 Table).

**Fig 6:**
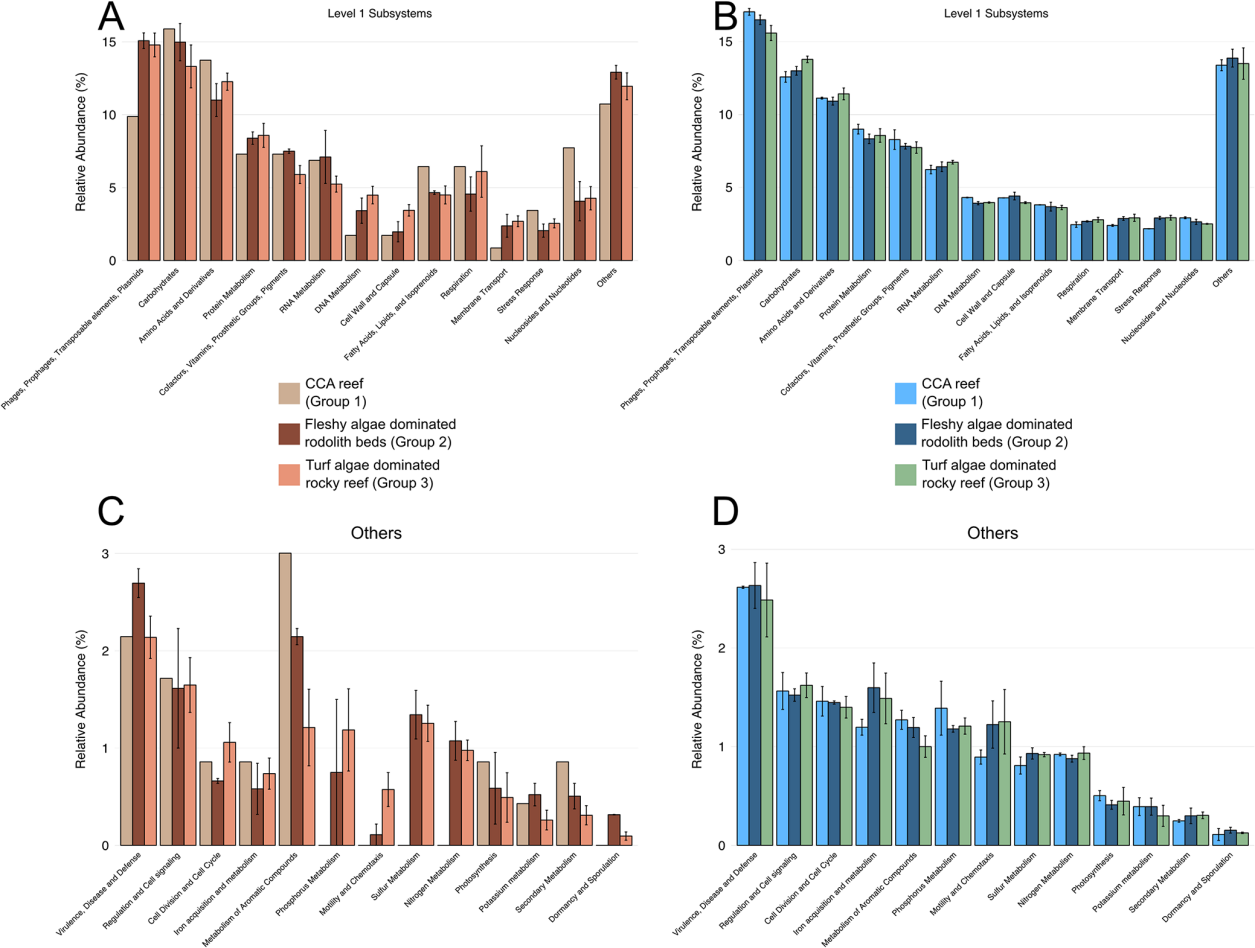
Functional diversity of the metagenomes. Relative abundance of the most frequent SEED Level 1 subsystems in coral (a) and water (b) samples. Relative abundance of the less abundant SEED Level 1 subsystems (“Others” category in plots a and b) in coral (c) and water (d) samples. Data are presented as the mean±SE.

Abundances of bacteria, Eukaryota and virus domains in water metagenomes were significantly different between the macro-habitat groups (ANOVA, *p*<0.05) (H3 confirmed) (S1 Fig and S4 and S5 Tables). Both rhodolith beds and rocky reefs (Groups 2 and 3, respectively) had higher abundances of bacterial sequences in water metagenomes compared with CCA reefs (Group 1) (S1 Fig). Eukaryota was significantly more abundant in the water metagenomes of CCA reefs than in those of rocky reefs and rhodolith beds (S1 Fig and S4 and S5 Tables). Among the eukaryotic phyla, Chlorophyta, Streptophyta, Arthropoda, Ascomycota, Nematoda, Cnidaria and Apicomplexa were significantly more abundant in CCA reef (Group 1) water metagenomes (Fig 5D, S1 Fig and S6 Table). Proteobacteria was the most common bacterial phylum in water metagenomes (Fig 5B). Among the bacterial phyla, Fibrobacteres, Bacteroidetes, Actinobacteria, Verrucomicrobia, Planctomycetes and Chlorobi were significantly different in the water metagenomes of macro-habitat groups (Fig 5B, S1 Fig and S6 Table). No significant differences between the subsystems from water of different macro-habitat groups were observed (H4 refuted). However, phages, prophages, transposable elements, and plasmids were more common in the water from CCA reefs (3.5%±0.4%, average±standard error) and rhodolith beds (2.9%±0.6%) (Group 1 and 2) compared with rocky reefs (1.5%±0.06%) (Fig 6D). There were no significant differences between bacterial or eukaryotic phyla and subsystems among corals from different macro-habitat groups (both H5 and H6 refuted). However, Bacteroidetes (bacteria) and Ascomycota (Eukaryota) were more common in rocky reefs (Fig 5A and 5C). The genes related to fast bacterial growth (e.g., respiration, DNA metabolism, protein metabolism, cell wall and capsule, membrane transport, nucleosides and nucleotides, motility and chemotaxis) were also more common in the corals from CCA reefs and rocky reefs (Group 1 and 2) (Fig 6).

**Fig 7:**
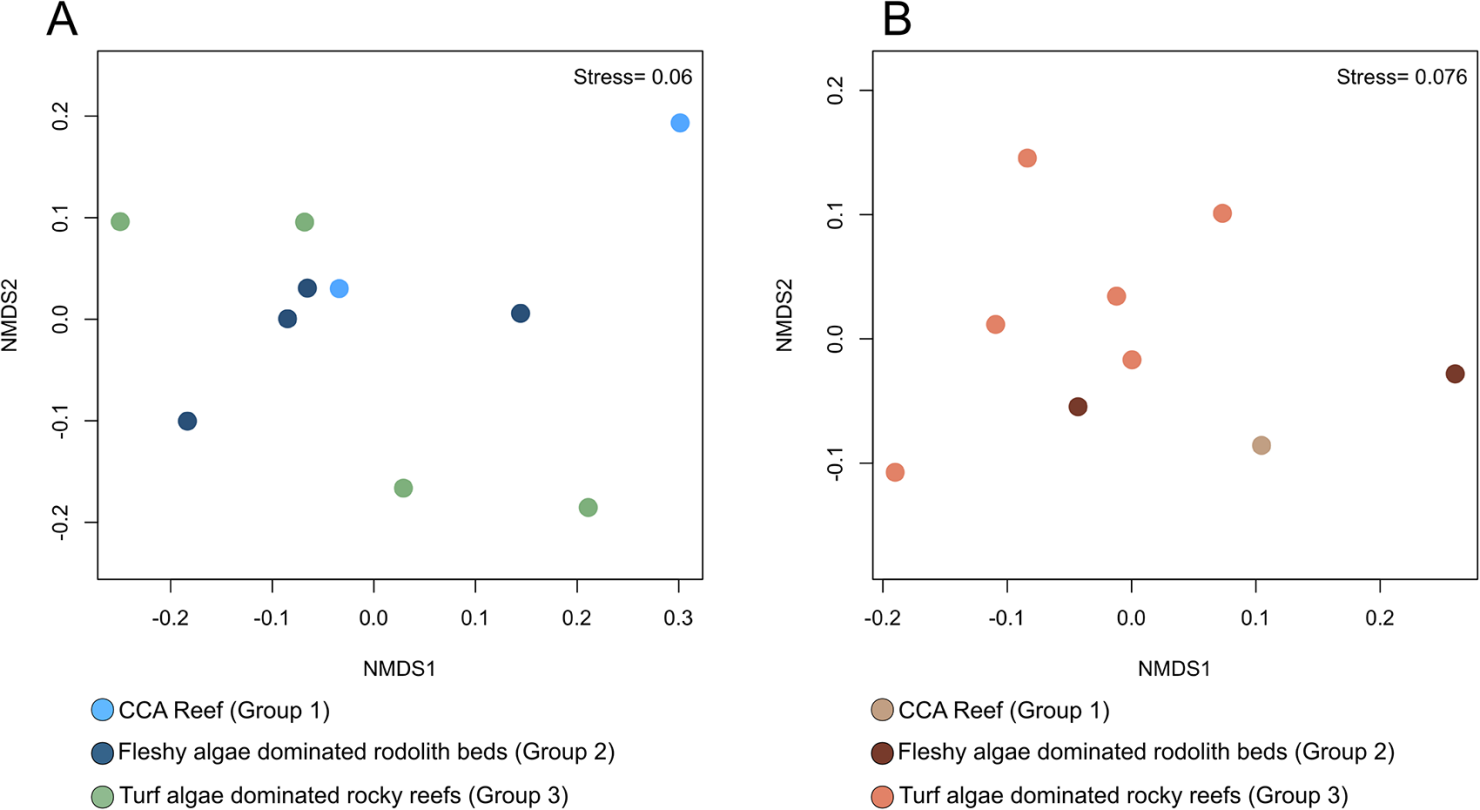
Nonmetric multidimensional scaling of dinucleotide signatures of water (a) and coral (b) metagenomes. There was no clear distinction between the samples, indicating that there is a genetic connectivity between the reef environments.

Based on the results of the NMDS using the calculated dinucleotide frequencies (Karlin’s signature) of the water and coral metagenomes, a clear distinction in these metagenomes was not observed between the different locations, indicating genetic connectivity (Fig 7A and 7B). There was no significant difference in the dinucleotide composition between the environmental groups (water microbiomes and corals from Groups 1, 2 and 3) based on results of the PERMANOVA (H7 refuted) (S7 and S8 Tables).

## Discussion

Results from the benthic surveys and ROV and diver recordings combined with side-scan sonar data indicate that the VTC represents a complex chain of rhodolith beds and consolidated reefs. Macro-habitat grouping was based primarily on quantitative benthic cover complemented by side-scan sonar data and diving observations.

### Benthic cover and irradiance may influence the health status of VTC corals

Corals from turf algae dominated rocky reefs (Group 3) showed visual signs of compromised health (e.g., tissue color anomalies and thin fleshy tissue with detectable lesions) in more than 90% of the examined coral colonies. However, colonies from CCA reefs (Group 1) generally presented visual signs of better health (e.g., homogeneous darker brownish-green color and thick tissue with signs of coral bleaching). *M*. *hispida* colonies from fleshy algae dominated rhodolith beds (Group 2) were clearly healthier because of their thicker tissue layer and greater tissue pigmentation. The above-mentioned conclusions on coral health status were supported by the results of the metagenomic analyses. Bacteroidetes (bacteria) and Ascomycota (Eukaryota) were more common in rocky reef corals. Both phyla were found in white plague diseased *Mussismilia brasiliensis* from Abrolhos Bank [34]. We also found a higher frequency of genes related to fast microbial growth in corals from the turf algae dominated rocky reefs and CCA reefs (Groups 3 and 1).

The additive effects of high irradiance stress and high DOC concentrations, probably exuded by turf algae, may be the cause of the high frequency of unhealthy corals at the turf algae dominated rocky reefs (Group 3). It is known that turf algae are a rich source of toxic microbes, implying that the turf algae produced DOC could be rich in toxic compounds that would negatively affect corals [45]. Microbial community composition associated with turf and fleshy algae are indeed different [46]. Turf algae exudates also increases microbial oxygen demand and lead to coral hypoxia [47]. In addition, light stress can lead to coral bleaching and faster disease progression [48–50]. In deeper sites (e.g. Groups 1 and 2) light is attenuated and can prevent coral disease occurrence [51]. In coastal environments, *M*. *hispida* is typically found on reef walls (low light and/or turbid water) [25] and is relatively common in mesophotic environments of the VTC and Trindade Island [25, 52]. Higher abundances of Gammaproteobacteria, a group containing fast-growing heterotrophic bacteria, in the water metagenomes of the turf algae dominated rocky reefs may be a result of the influence of turf algae exudates. The bacterial activity, stimulated by the high DOC concentrations, may act together with the high irradiance increasing coral disease at Euphotic reefs in VTC (Group 3). The results presented here reinforce the possible ecological interactions among the benthos (e.g. *Mussismilia* corals), abiotic factors (e.g. depth and irradiance), fish communities, nutrients and microbiome in reef systems of VTC as seen in other reef systems [33].

Corals found during our benthic surveys at VTC were also abundant at Abrolhos Bank (e.g., broadcasting-spawning corals *M*. *cavernosa* and *M*. *hispida*) [25]. Studies have shown that certain broadcasting-spawning corals from the Atlantic Ocean (including Brazilian corals) can disperse over 3,000 km [53, 54]. However, more recent studies have shown that *M*. *cavernosa* populations are structured by depth, indicating low population connectivity [4, 55]. We observed genetic connectivity in water microbiomes and *M*. *hispida* holobionts based on their dinucleotide frequencies and the taxonomic and functional composition of coral metagenomes. However, it is clear that the benthic and fish communities are distributed across at least three major groups, which suggests a certain level of isolation between seamount communities and the need to consider each seamount/group as a distinct system. Because of the broad bathymetrical distribution of *M*. *hispida* across the euphotic-mesophotic gradient, populations of *M*. *hispida* within mesophotic rhodolith beds at VTC could play a critical role in the survival of *M*. *hispida* populations in the face of global changes and increased human impacts.

The results of our fish assemblage surveys indicate that rocky reefs around Trindade Island are impacted environments. The reduction of large and carnivorous fish biomass observed in the rocky reefs at Trindade Island may be a result of fishing activity, which can reduce the biomass of commercially important large carnivores (e.g., blue sharks, nurse sharks, reef sharks and yellowfin groupers) [14, 56]. A high biomass of large and carnivorous fish was observed at the CCA reefs on Davis Seamount. A high abundance of macro-carnivores and large fish is a characteristic of healthy oceanic and coastal coral reef environments [33, 57]. CCA reefs (Group 1) showed high habitat complexity. Habitat complexity from other oceanic provinces is related to important coral reef features, including enhanced herbivory of reef fish; increased fish density, richness, diversity, abundance and biomass; and decreased algal cover [58–60]. Habitat complexity also increases foraging activity of two species of Brazilian rocky reef wrasses [61]. Previous studies in the VTC region also support our findings and indicate that the biomass of herbivorous and carnivorous fish were higher in the CCA reefs than in the rocky reefs surrounding Trindade Island [56].

### VTC may influence planktonic microbial abundance and productivity

The VTC has been identified as a hotspot of microbial abundance and productivity [12, 13]. However, possible effects of benthic-pelagic coupling in the VTC seamounts on the pelagic microbial abundance and diversity have not been previously evaluated. Rhodolith beds (Group 2) had a high microbial abundance, richness and diversity. These variables were highly correlated with primary productivity proxies (chlorophyll *a* and phaeophytin), which may indicate benthic-pelagic coupling effects of seamounts in their surrounding pelagic compartment. The observed high frequency of eukaryote and virus metagenomic sequences may indicate a high dynamic lytic viral cycling at these sites. Therefore, the viral populations may be actively lysing the host cells and releasing organic matter in the water, which would increase its availability for heterotrophic bacteria [62]. Highly dynamic lytic viral cycling may contribute to higher bacterial abundance in rhodolith bed sites.

**Fig 8:**
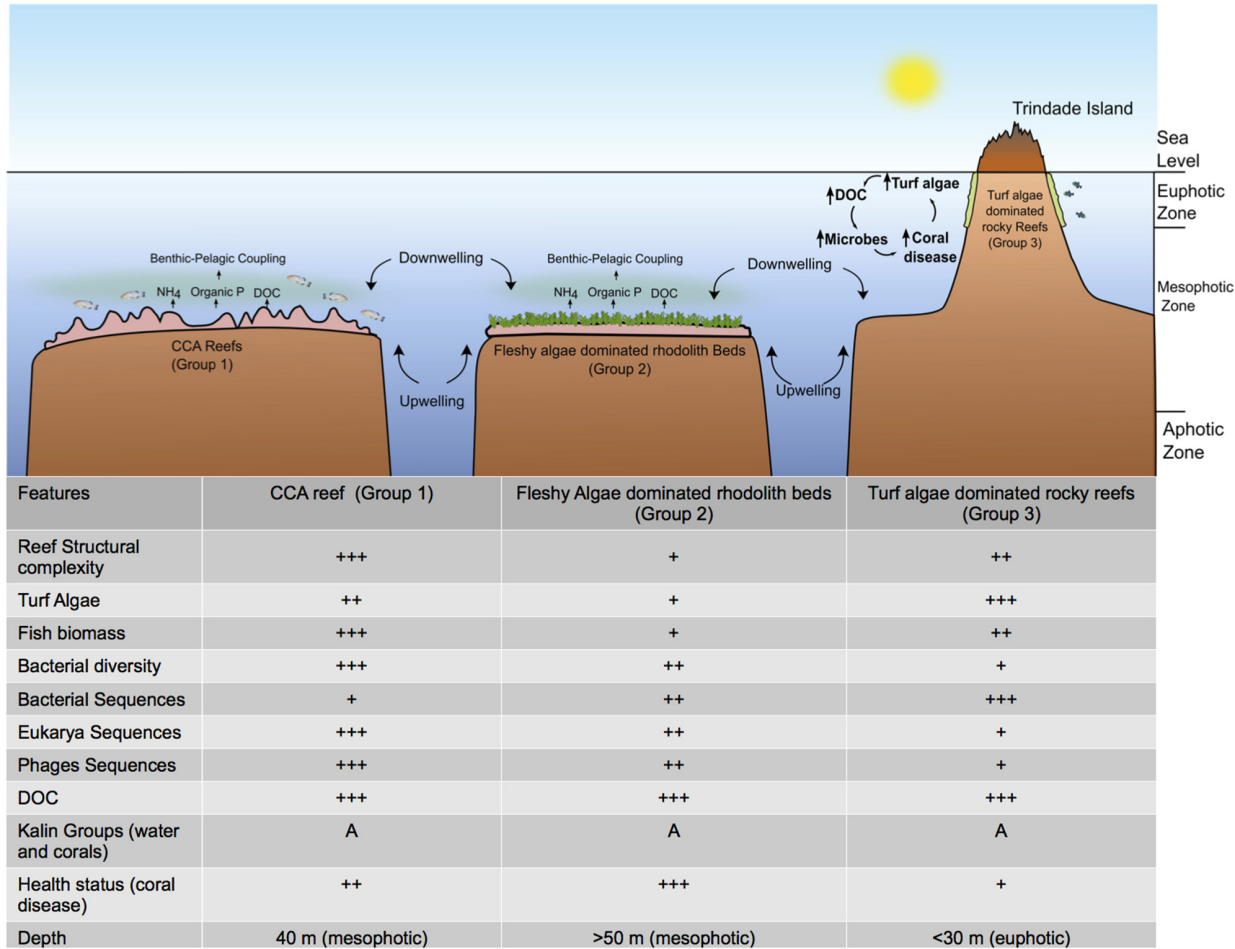
Hypothetical schematic representation of VTC macro-habitats and processes. CCA reefs (Group 1), fleshy algae dominated rhodolith beds (Group 2) and turf algae dominated rocky reefs (Group 3) are represented. In pink, CCA reefs and fleshy algae (dark green) dominated rhodolith beds. In light green, high turf algae cover at rocky reefs. The highly complex habitat of CCA reefs harbors high biomass of carnivorous fishes. Input of nutrients and upwelling/downwelling generating microbial productivity. During downwelling periods, regenerated and/or excreted nutrients (e.g., ammonia and phosphorus) may be provided to the system by the benthic organisms/microorganisms. At Group 3 Euphotic reefs microbial growth stimulated by DOC released by turf algae may have toxic effects and cause coral disease. At Group 1 and 2 mesophotic environments probably fleshly algae is the major DOC producer, having no toxic effects on coral health. During upwelling periods, new nutrients (nitrate) from deeper water masses may become available to the systems. Microbial communities are connected between the seamounts (Karlin signature). Not to scale. Grouper and black durgon pictures from www.fishbase.org [29].

The benthic influence on water column microbes may occur via translocation of excreted nutrients (e.g., DOC and ammonia) and mineralization by benthic organisms [63]. The estimated area covered by rhodolith beds on the top of the VTC seamounts is 85,937 km^2^ [19]. Assuming that VTC rhodoliths have a similar photosynthetic potential (52.16 μmol carbon m^-2^.s^-1^) to those on the Abrolhos Bank [64], the VTC would produce approximately 23.6x10^3^ tons of DOC per day. The impact of the VTC in the fertilization of the surrounding waters with DOC is clear and may lead to increased pelagic microbial abundance and growth, particularly during downweling periods when nutrients coming from deep waters may be depleted. Planktonic primary and bacterial productivity may influence benthic communities because once the biomass of lower trophic levels (e.g., meiofauna) increases, the higher trophic level biomass tends to also increase (e.g., invertivorous and carnivorous fish) [5]. In addition, seamounts may promote the formation of upwelling and downwelling regions. These processes increase nutrient concentrations over seamount peaks via different mechanisms; consequently, primary and bacterial production also increases [65]. During our study, clear evidence of upwelling was not observed because the nutrient concentrations (e.g., nitrate concentration) were low. However, downwelling and benthic pelagic coupling may have occurred during our study (Fig 8). During downwelling periods, the inorganic nutrient concentration of the water column is low and exudates and excreted nutrients of benthic organisms may help microbes grow in the surrounding waters. DOC concentration in all locations of this study was high (approx. 200 μM), more than twice the values found in South Atlantic reefs [33] and higher than found in Campos Basin [66], suggesting high levels of productivity in VTC seamounts.

### Davis Seamount as a candidate for a new non-take marine protected area

We argue for the establishment of a non-take protected area for several reasons. First, this is a unique environment within the VTC, and this seamount extends throughout the shallowest portion at the end of the seamount chain from west to east and possibly represents the final stepping-stone for connectivity between the continental shelf and Trindade Island. Davis Seamount contains many shallow-water herbivorous populations, such as *Acanthurus bahianus*, *Microspathodon chrysurus*, *Ophioblennius trinitatis*, *Scarus zelindae* and *Stegastes fuscus*. This seamount also has one of the largest summit areas of the VTC, a feature that may amplify its resilience and support capacity, and it also embraces one of the largest rhodolith beds of the entire VTC. Thus, Davis Seamount is a large CaCO_3_ reservoir and functions as a seedbed for marine life, promoting the life stages of different types of (in)vertebrates [64]. This critically important role has been underestimated in the past, but we now know that rhodoliths harbor great biodiversity. The biological productivity of DOC by rhodoliths plays a significant ecological role, which has been previously demonstrated for the Abrolhos Bank [67].

This is the first study characterizing the VTC environments by means of a holistic approach involving water chemistry, microbial abundance and diversity, and benthic and fish surveys. The integrated analyses of different datasets demonstrates that heterogeneous systems of the VTC seamounts sustain high biodiversity and play important ecological roles in the health maintenance of the southwestern Atlantic Ocean. This study also indicates that VTC seamounts may have direct effects on the abundance and diversity of the surrounding water column microbes and coral health. The study hypotheses 1 to 3 were confirmed, while the hypotheses 4 to 7 were refuted. The VTC mounts can thus be considered different systems, with some degree of genetic connectivity. We recorded three major reef macro-habitats: 1) CCA reefs at Davis Seamount; 2) mesophotic fleshy algae dominated rhodolith beds; and 3) turf algae dominated rocky reefs at Trindade Island. Each of these macro-habitats has a typical characteristic for benthic cover, fish composition and biomass and microbial community diversity. We also revealed benthic-pelagic coupling features, including nutrient (e.g., ammonia and silicate) input from the benthic compartment into the pelagic compartment. Massive DOC production may also influence water column microbial abundance and diversity. This study emphasizes the ecological and economic importance of VTC as a resource for biodiversity, fisheries and mineral mining. There is an urgent need to establish a large marine protected area that encompasses the diversity of reef systems in the VTC because they may play fundamental ecological roles in the health of the southwestern Atlantic Ocean.

## Supporting Information

S1 Fig STAMP graphical illustration of ANOVA Tukey-Kramer post-hoc tests. Only domains or phyla with significant differences (corrected *p*<0.05) were included in this figure.
(DOCX)

S1 Table Number of samples per site. (DOCX)

S2 Table General features of the metagenomes. (DOCX)

S3 Table Adonis (PERMANOVA) results of benthic cover based on Bray-Curtis distances with 999 permutations. MS, mean sum of squares; SS, sum of squares.
(DOCX)

S4 Table Metagenomes taxonomic annotation at the domain level and bacterial family diversity. (DOCX)

S5 Table Water metagenomes phyla with significant difference between the environmental groups. ANOVA; eta-squared effect size statistics and Storey false discovery rate correction for multiple tests.
(DOCX)

S6 Table Water metagenomes phyla with significant difference between the environmental groups. ANOVA; eta-squared effect size statistics and Storey false discovery rate correction for multiple tests.
(DOCX)

S7 Table Adonis (PERMANOVA) results of the dinucleotide analysis of coral metagenomes based on Bray-Curtis distances with 999 permutations. MS, mean sum of squares; SS, sum of squares.
(DOCX)

S8 Table Adonis (PERMANOVA) results for the dinucleotide analysis of coral metagenomes based on Bray-Curtis distances with 999 permutations. MS, mean sum of squares; SS, sum of squares.
(DOCX)
