# Supplementary material for: Baseline Assessment of Mesophotic Reefs of the Vitória-Trindade Seamount Chain Based on Water Quality, Microbial Diversity, Benthic Cover and Fish Biomass Data
Source: PLoS One. 2015 Jun 19;10(6):e0130084. doi: 10.1371/journal.pone.0130084 (PMC4474894; doi:10.1371/journal.pone.0130084)
Supplement: S1 Fig — Only domains or phyla with significant differences (corrected p<0.05) were included in this figure. (DOCX) [file pone.0130084.s001.docx]

**
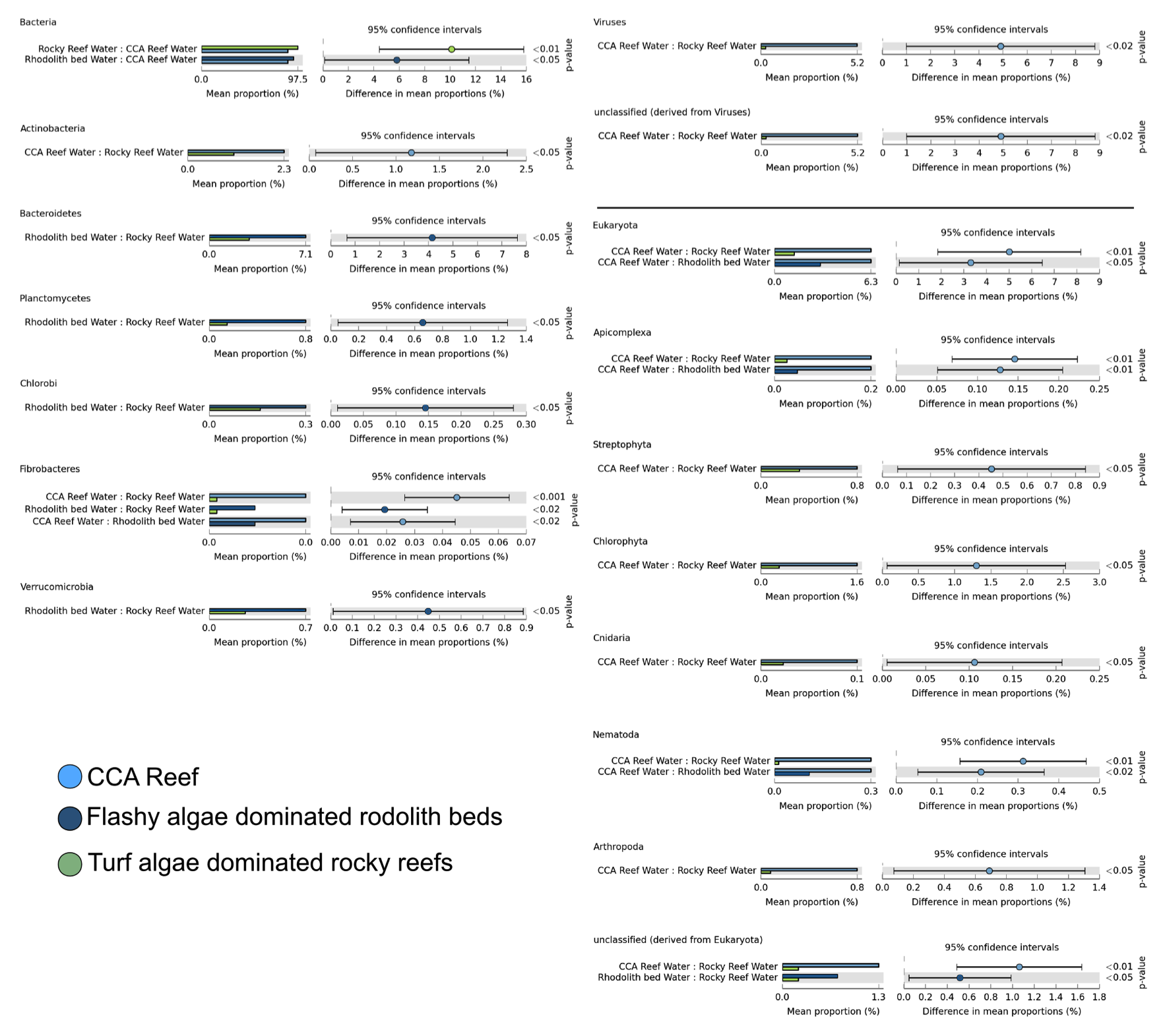
**

**S1 Fig. – STAMP graphical illustration of ANOVA Turkey-Kramer pos-hoc tests.** Only domains or phyla with significant differences (corrected *p*<0.05) were included in this figure.
