# Supplementary material for: Baseline Assessment of Mesophotic Reefs of the Vitória-Trindade Seamount Chain Based on Water Quality, Microbial Diversity, Benthic Cover and Fish Biomass Data
Source: PLoS One. 2015 Jun 19;10(6):e0130084. doi: 10.1371/journal.pone.0130084 (PMC4474894; doi:10.1371/journal.pone.0130084)
Supplement: S1 Table — (DOCX) [file pone.0130084.s002.docx]

**S1 Table – Number of samples per site.**

| Sampling site | Collection Date (dd/mm/yy) | Depth (m) | Benthic Photoquadrats | Fish (video frames) | Water metagenomic samples | Coral metagenomic samples (Specimen Number) ^1^ | Nutrients | Bacterial counts |
| --- | --- | --- | --- | --- | --- | --- | --- | --- |
| Eclaireur | 07/02/11 | 71 | 16 |  |  |  | 3 | 4 |
| Jaseur | 09/02/11 | 60 | 28 | 91 |  | 1 (CVT#01) |  |  |
| Columbia Bank | 16/02/11 | 62 | 10 |  |  |  |  |  |
| Almirante Saldanha | 24/02/11 | 66 | 13 |  |  |  |  |  |
| Vitoria | 04/02/11 | 63 |  |  | 2 | 1 (CVT#02) | 3 | 4 |
| Davis | 11/02/11 | 40 | 11 | 275 | 2 | 1 (CVT#03) | 3 | 4 |
| Trindade Shelf | 18/02/11 | 50 | 10 |  | 2 |  | 3 | 4 |
| Enseada do Principe | 16/03/09 | 20 | 10 |  | 2 |  | 3 |  |
| Ponta Noroeste | 18/03/09 | 15 | 9 |  |  |  | 3 |  |
| Ponta Noroeste | 19/03/09 | 20 | 10 |  | 2 | 3 (CVT#04-06) | 3 |  |
| Ponta dos Farilhoes | 21/03/09 and 18/02/11 | 22 | 10 | 180 |  | 3 (CVT#07-09) | 3 | 4 |

**^1 –^** Specimen number on collection of the [Laboratory of Microbiology](http://www.microbiologia.biologia.ufrj.br) at Federal University of Rio de Janeiro.
