# Supplementary material for: Baseline Assessment of Mesophotic Reefs of the Vitória-Trindade Seamount Chain Based on Water Quality, Microbial Diversity, Benthic Cover and Fish Biomass Data
Source: PLoS One. 2015 Jun 19;10(6):e0130084. doi: 10.1371/journal.pone.0130084 (PMC4474894; doi:10.1371/journal.pone.0130084)
Supplement: S2 Table — (DOCX) [file pone.0130084.s003.docx]

**S2 Table – General features of the metagenomes.**

| **MG RAST ID** | **Sample Name** | **Source** | **Site Name** | **Location** | **Habtat** | **# of Reads** | **Read Length Average (bp)** | **Reads Length STD (bp)** | **Average GC Content (%)** | **Identified Sequences (Taxonomic) (%)** | **Identified Sequences (Functional) (%)** |
| --- | --- | --- | --- | --- | --- | --- | --- | --- | --- | --- | --- |
| 4480748.3 | DAV_Mount_W1 | Water | Davis | Davis Seamout | CCA Reef | 13623 | 345.49 | 122.3 | 45.66 | 4772 (35.03) | 6739 (49.47) |
| 4480749.3 | DAV_Mount_W2 | Water | Davis | Davis Seamout | CCA Reef | 16365 | 370.22 | 115.75 | 44.08 | 7862 (48.04) | 11218 (68.55) |
| 4484839.3 | DAV_Mount_C | Coral | Davis | Davis Seamout | CCA Reef | 40485 | 307.21 | 123.07 | 40.63 | 1534 (3.79) | 255 (0.63) |
| 4487651.3 | PRI_Island_W1 | Water | Enseada do Príncipe | Trindade Island | Rocky Reef | 12844 | 287.88 | 120.15 | 37.78 | 6743 (52.5) | 10953 (85.28) |
| 4487647.3 | PRI_Island_W3 | Water | Enseada do Príncipe | Trindade Island | Rocky Reef | 22552 | 432.11 | 109.26 | 36.89 | 16167 (71.69) | 26219 (116.26) |
| 4480741.3 | FAR_Island_C1 | Coral | Enseada dos 5 Farilhões | Trindade Island | Rocky Reef | 24714 | 369.71 | 109.74 | 39.39 | 1213 (4.91) | 198 (0.8) |
| 4480742.3 | FAR_Island_C2 | Coral | Enseada dos 5 Farilhões | Trindade Island | Rocky Reef | 26258 | 367.64 | 114.51 | 42.93 | 3709 (14.13) | 3628 (13.82) |
| 4480743.3 | FAR_Island_C3 | Coral | Enseada dos 5 Farilhões | Trindade Island | Rocky Reef | 36216 | 370.94 | 123.98 | 39.28 | 1938 (5.35) | 328 (0.91) |
| 4480740.3 | JAS_Mout_C | Coral | Jaseur | Jaseur Seamount | Rhodolith Bed | 31860 | 360.04 | 108.25 | 39.38 | 1434 (4.5) | 360 (1.13) |
| 4487649.3 | NOR_Island_W1 | Water | Ponta Noroeste | Trindade Island | Rocky Reef | 8182 | 288.73 | 117.68 | 39.11 | 4397 (53.74) | 6691 (81.78) |
| 4487650.3 | NOR_Island_W2 | Water | Ponta Noroeste | Trindade Island | Rocky Reef | 15111 | 214.68 | 91.17 | 41.09 | 9153 (60.57) | 12998 (86.02) |
| 4487911.3 | NOR_Island_C3 | Coral | Ponta Noroeste | Trindade Island | Rocky Reef | 28781 | 455.39 | 93.26 | 52.29 | 8282 (28.78) | 9275 (32.23) |
| 4487909.3 | NOR_Island_C1 | Coral | Ponta Noroeste | Trindade Island | Rocky Reef | 41409 | 451.82 | 109.66 | 42.82 | 4626 (11.17) | 1760 (4.25) |
| 4487910.3 | NOR_Island_C2 | Coral | Ponta Noroeste | Trindade Island | Rocky Reef | 90559 | 439.13 | 128.3 | 40.91 | 7614 (8.4) | 4165 (4.6) |
| 4480750.3 | SAN_Island_W1 | Water | Trindade Shelf | Trindade Island | Rocky Reef | 15920 | 376.27 | 111.96 | 44.02 | 7702 (48.38) | 11538 (72.47) |
| 4480751.3 | SAN_Island_W2 | Water | Trindade Shelf | Trindade Island | Rocky Reef | 20206 | 398.97 | 105.53 | 43.5 | 10469 (51.81) | 15242 (75.43) |
| 4480746.3 | VIT_Mout_W1 | Water | Vitoria | Vitoria Seamount | Rhodolith Bed | 11368 | 392.7 | 99.69 | 44.96 | 7012 (61.68) | 9741 (85.69) |
| 4480747.3 | VIT_Mout_W2 | Water | Vitoria | Vitoria Seamount | Rhodolith Bed | 18438 | 398.49 | 98.43 | 42.84 | 10591 (57.44) | 15318 (83.08) |
| 4480739.3 | VIT_Mount_C | Coral | Vitoria | Vitoria Seamount | Rhodolith Bed | 26181 | 309.36 | 112.95 | 45.25 | 3405 (13.01) | 3488 (13.32) |
