# Supplementary material for: Baseline Assessment of Mesophotic Reefs of the Vitória-Trindade Seamount Chain Based on Water Quality, Microbial Diversity, Benthic Cover and Fish Biomass Data
Source: PLoS One. 2015 Jun 19;10(6):e0130084. doi: 10.1371/journal.pone.0130084 (PMC4474894; doi:10.1371/journal.pone.0130084)
Supplement: S3 Table — MS, mean sum of squares; SS, sum of squares. (DOCX) [file pone.0130084.s004.docx]

**S3Table – Adonis (Permanova) results of benthic cover based on Bray-Curtis distances with 999 permutations. MS, mean sum of squares; SS, sum of squares.**

|  | d.f. | SS | MS | pseudoF | R^2^ | P-Value |
| --- | --- | --- | --- | --- | --- | --- |
| Environment groups | 2 | 7.68 | 3.68 | 19.37 | 0.24 | 0.001 |
| Residuals | 121 | 22.97 | 0.19 |  | 0.76 |  |
| Total | 123 | 30.32 |  |  | 1 |  |
