# Supplementary material for: Baseline Assessment of Mesophotic Reefs of the Vitória-Trindade Seamount Chain Based on Water Quality, Microbial Diversity, Benthic Cover and Fish Biomass Data
Source: PLoS One. 2015 Jun 19;10(6):e0130084. doi: 10.1371/journal.pone.0130084 (PMC4474894; doi:10.1371/journal.pone.0130084)
Supplement: S4 Table — (DOCX) [file pone.0130084.s005.docx]

**S4 Table – Metagenomes taxonomic annotation at Domain level and bacterial family diversity.**

| metagenome | Sample Name | Source | Bacteria (%) | Eukaryota (%) | Viruses (%) | Archaea (%) | unclassified sequences (%) | other sequences (%) | Unassigned (%) | Bacterial families Richness | annon entropy | Shannon Evenness |
| --- | --- | --- | --- | --- | --- | --- | --- | --- | --- | --- | --- | --- |
| 4480747.3 | VIT_Mout_W2 | Water | 9499 (89.69) | 332 (3.13) | 665 (6.28) | 32 (0.3) | 50 (0.47) | 13 (0.12) | 0 (0) | 217 | 3.835 | 0.213 |
| 4480746.3 | VIT_Mout_W1 | Water | 6617 (94.37) | 134 (1.91) | 220 (3.14) | 20 (0.29) | 21 (0.3) | 0 (0) | 0 (0) | 198 | 3.767 | 0.218 |
| 4480751.3 | SAN_Island_W2 | Water | 9874 (94.32) | 307 (2.93) | 158 (1.51) | 43 (0.41) | 78 (0.75) | 8 (0.08) | 1 (0.01) | 231 | 4.025 | 0.242 |
| 4480750.3 | SAN_Island_W1 | Water | 7257 (94.22) | 288 (3.74) | 71 (0.92) | 41 (0.53) | 36 (0.47) | 9 (0.12) | 0 (0) | 220 | 4.013 | 0.251 |
| 4487647.3 | PRI_Island_W3 | Water | 15584 (96.39) | 316 (1.95) | 34 (0.21) | 65 (0.4) | 162 (1) | 3 (0.02) | 3 (0.02) | 236 | 3.641 | 0.162 |
| 4487651.3 | PRI_Island_W1 | Water | 6534 (96.9) | 108 (1.6) | 23 (0.34) | 28 (0.42) | 49 (0.73) | 1 (0.01) | 0 (0) | 200 | 3.465 | 0.16 |
| 4487650.3 | NOR_Island_W2 | Water | 9047 (98.84) | 42 (0.46) | 12 (0.13) | 16 (0.17) | 27 (0.29) | 9 (0.1) | 0 (0) | 179 | 3.123 | 0.127 |
| 4487649.3 | NOR_Island_W1 | Water | 4320 (98.25) | 42 (0.96) | 13 (0.3) | 5 (0.11) | 14 (0.32) | 3 (0.07) | 0 (0) | 170 | 3.439 | 0.183 |
| 4480749.3 | DAV_Mount_W2 | Water | 7085 (90.12) | 330 (4.2) | 358 (4.55) | 44 (0.56) | 41 (0.52) | 3 (0.04) | 1 (0.01) | 211 | 3.85 | 0.223 |
| 4480748.3 | DAV_Mount_W1 | Water | 3998 (83.78) | 400 (8.38) | 321 (6.73) | 22 (0.46) | 30 (0.63) | 0 (0) | 1 (0.02) | 197 | 3.813 | 0.23 |
| 4480739.3 | VIT_Mount_C | Coral | 2662 (78.18) | 669 (19.65) | 3 (0.09) | 43 (1.26) | 10 (0.29) | 16 (0.47) | 2 (0.06) | 216 | 4.739 | 0.529 |
| 4487911.3 | NOR_Island_C3 | Coral | 7435 (89.77) | 615 (7.43) | 0 (0) | 193 (2.33) | 26 (0.31) | 10 (0.12) | 3 (0.04) | 256 | 4.817 | 0.483 |
| 4487910.3 | NOR_Island_C2 | Coral | 2823 (37.08) | 4648 (61.05) | 2 (0.03) | 51 (0.67) | 5 (0.07) | 72 (0.95) | 13 (0.17) | 213 | 4.68 | 0.506 |
| 4487909.3 | NOR_Island_C1 | Coral | 1201 (25.96) | 3343 (72.27) | 3 (0.06) | 43 (0.93) | 6 (0.13) | 30 (0.65) | 0 (0) | 180 | 4.633 | 0.571 |
| 4480740.3 | JAS_Mout_C | Coral | 110 (7.67) | 1299 (90.59) | 0 (0) | 5 (0.35) | 0 (0) | 18 (1.26) | 2 (0.14) | 53 | 3.733 | 0.789 |
| 4480743.3 | FAR_Island_C3 | Coral | 96 (4.95) | 1801 (92.93) | 1 (0.05) | 6 (0.31) | 0 (0) | 33 (1.7) | 1 (0.05) | 57 | 3.855 | 0.829 |
| 4480742.3 | FAR_Island_C2 | Coral | 2737 (73.79) | 785 (21.16) | 5 (0.13) | 151 (4.07) | 10 (0.27) | 21 (0.57) | 0 (0) | 214 | 4.677 | 0.502 |
| 4480741.3 | FAR_Island_C1 | Coral | 83 (6.84) | 1102 (90.85) | 0 (0) | 4 (0.33) | 0 (0) | 24 (1.98) | 0 (0) | 37 | 3.256 | 0.701 |
| 4484839.3 | DAV_Mount_C | Coral | 112 (7.3) | 1390 (90.61) | 1 (0.07) | 2 (0.13) | 0 (0) | 28 (1.83) | 1 (0.07) | 51 | 3.68 | 0.778 |
