# Supplementary material for: Baseline Assessment of Mesophotic Reefs of the Vitória-Trindade Seamount Chain Based on Water Quality, Microbial Diversity, Benthic Cover and Fish Biomass Data
Source: PLoS One. 2015 Jun 19;10(6):e0130084. doi: 10.1371/journal.pone.0130084 (PMC4474894; doi:10.1371/journal.pone.0130084)
Supplement: S5 Table — ANOVA; eta-squared effect size statistics and Storey false discovery rate correction for multiple tests. (DOCX) [file pone.0130084.s006.docx]

**S5 Table – Water metagenomes Domains with significant difference between the environmental Groups. ANOVA; Eta-squared effect size statistics and Storey False Discovery Rate correction for multiple tests.**

| Domain | P-values | P-values (corrected) | Effect size |
| --- | --- | --- | --- |
| Bacteria | 0.004 | 0.025 | 0.800 |
| Eukaryota | 0.007 | 0.025 | 0.757 |
| Viruses | 0.020 | 0.046 | 0.675 |
