# Supplementary material for: Baseline Assessment of Mesophotic Reefs of the Vitória-Trindade Seamount Chain Based on Water Quality, Microbial Diversity, Benthic Cover and Fish Biomass Data
Source: PLoS One. 2015 Jun 19;10(6):e0130084. doi: 10.1371/journal.pone.0130084 (PMC4474894; doi:10.1371/journal.pone.0130084)
Supplement: S6 Table — ANOVA; eta-squared effect size statistics and Storey false discovery rate correction for multiple tests. (DOCX) [file pone.0130084.s007.docx]

**S6 Table – Water metagenomes Phyla with significant difference between the environmental Groups. ANOVA; Eta-squared effect size statistics and Storey False Discovery Rate correction for multiple tests.**

| Domain | Phylum | P-values | P-values (corrected) | Effect size |
| --- | --- | --- | --- | --- |
| Archaea | unclassified (derived from Archaea) | 0.001 | 0.005 | 0.846 |
| Bacteria | Fibrobacteres | 0.001 | 0.005 | 0.880 |
| Bacteria | Bacteroidetes | 0.025 | 0.027 | 0.650 |
| Bacteria | Actinobacteria | 0.029 | 0.027 | 0.637 |
| Bacteria | Verrucomicrobia | 0.029 | 0.027 | 0.635 |
| Bacteria | Planctomycetes | 0.036 | 0.027 | 0.614 |
| Bacteria | Chlorobi | 0.037 | 0.027 | 0.609 |
| Eukaryota | Nematoda | 0.002 | 0.005 | 0.834 |
| Eukaryota | Apicomplexa | 0.002 | 0.005 | 0.828 |
| Eukaryota | unclassified (derived from Eukaryota) | 0.003 | 0.005 | 0.815 |
| Eukaryota | Streptophyta | 0.032 | 0.027 | 0.626 |
| Eukaryota | Arthropoda | 0.036 | 0.027 | 0.614 |
| Eukaryota | Chlorophyta | 0.037 | 0.027 | 0.611 |
| Eukaryota | Cnidaria | 0.048 | 0.032 | 0.580 |
| Eukaryota | Ascomycota | 0.051 | 0.032 | 0.574 |
| Eukaryota | Chordata | 0.062 | 0.036 | 0.548 |
| Viruses | unclassified (derived from Viruses) | 0.020 | 0.027 | 0.675 |
