# Supplementary material for: Baseline Assessment of Mesophotic Reefs of the Vitória-Trindade Seamount Chain Based on Water Quality, Microbial Diversity, Benthic Cover and Fish Biomass Data
Source: PLoS One. 2015 Jun 19;10(6):e0130084. doi: 10.1371/journal.pone.0130084 (PMC4474894; doi:10.1371/journal.pone.0130084)
Supplement: S8 Table — MS, mean sum of squares; SS, sum of squares. (DOCX) [file pone.0130084.s009.docx]

**S8 Table – Adonis (Permanova) results for dinucleotide analysis of coral metagenomes based on Bray-Curtis distances with 999 permutations. MS, mean sum of squares; SS, sum of squares.**

|  | d.f. | SS | MS | pseudoF | R^2^ | P-Value |
| --- | --- | --- | --- | --- | --- | --- |
| Environment groups | 2 | 0.03 | 0.02 | 1.22 | 0.29 | 0.303 |
| Residuals | 6 | 0.07 | 0.01 |  | 0.71 |  |
| Total | 8 | 0.10 |  |  | 1 |  |
